# Pharmacological Activity, Pharmacokinetics, and Toxicity of Timosaponin AIII, a Natural Product Isolated From *Anemarrhena asphodeloides* Bunge: A Review

**DOI:** 10.3389/fphar.2020.00764

**Published:** 2020-06-03

**Authors:** Yan Lin, Wai-Rong Zhao, Wen-Ting Shi, Jing Zhang, Kai-Yu Zhang, Qian Ding, Xin-Lin Chen, Jing-Yi Tang, Zhong-Yan Zhou

**Affiliations:** ^1^Department of Cardiovascular Research Laboratory, Longhua Hospital, Shanghai University of Traditional Chinese Medicine, Shanghai, China; ^2^Department of Oncology, The Fourth Affiliated Hospital of Xinjiang Medical University, Urumqi, China; ^3^College of Basic Medicine, Guizhou University of Traditional Chinese Medicine, Guiyang, China; ^4^State Key Laboratory of Quality Research in Chinese Medicine and Institute of Chinese Medical Sciences, University of Macau, Macao, Macau

**Keywords:** Timosaponin AIII, autophagy, apoptosis, angiogenesis, inflammation

## Abstract

*Anemarrhena asphodeloides* Bunge is a famous Chinese Materia Medica and has been used in traditional Chinese medicine for more than two thousand years. Steroidal saponins are important active components isolated from *A. asphodeloides* Bunge. Among which, the accumulation of numerous experimental studies involved in Timosaponin AIII (Timo AIII) draws our attention in the recent decades. In this review, we searched all the scientific literatures using the key word “timosaponin AIII” in the PubMed database update to March 2020. We comprehensively summarized the pharmacological activity, pharmacokinetics, and toxicity of Timo AIII. We found that Timo AIII presents multiple-pharmacological activities, such as anti-cancer, anti-neuronal disorders, anti-inflammation, anti-coagulant, and so on. And the anti-cancer effect of Timo AIII in various cancers, especially hepatocellular cancer and breast cancer, is supposed as its most potential activity. The anti-inflammatory activity of Timo AIII is also beneficial to many diseases. Moreover, VEGFR, X-linked inhibitor of apoptosis protein (XIAP), B-cell-specific Moloney murine leukemia virus integration site 1 (BMI1), thromboxane (Tx) A2 receptor, mTOR, NF-κB, COX-2, MMPs, acetylcholinesterase (AChE), and so on are identified as the crucial pharmacological targets of Timo AIII. Furthermore, the hepatotoxicity of Timo AIII was most concerned, and the pharmacokinetics and toxicity of Timo AIII need further studies in diverse animal models. In conclusion, Timo AIII is potent as a compound or leading compound for further drug development while still needs in-depth studies.

## Introduction

**Figure 1:**
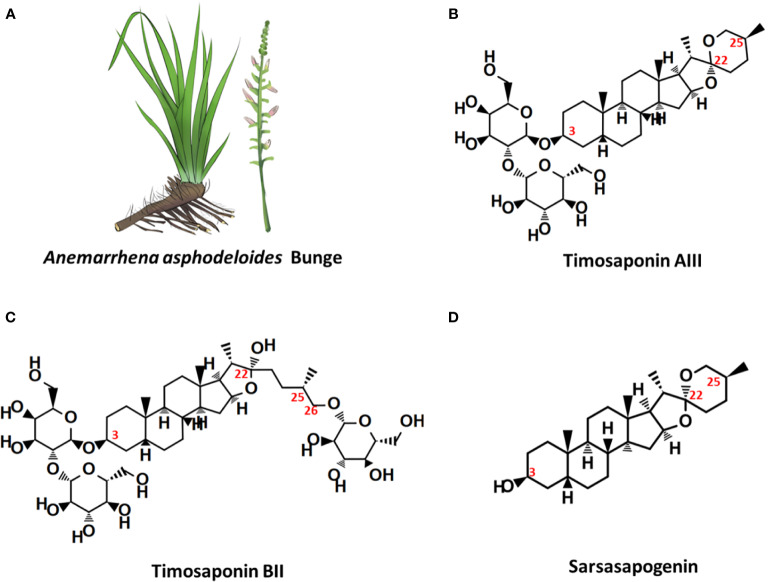
*Anemarrhena asphodeloides* Bunge and the chemical structures of its main steroidal saponin ingredients. **(A)** *A. asphodeloides* Bunge. **(B)** Timosaponin AIII, Pubchem CID: 71306914, MF: C_39_H_64_O_13_. **(C)** Timosaponin BII, Pubchem CID: 44575945, MF: C_45_H_76_O_19_. **(D)** Sarsasapogenin, Pubchem CID: 92095, MF: C_27_H_44_O_3_.

Timosaponin AIII (Timo AIII, IUPAC name: (2*S*,3*R*,4*S*,5*S*,6*R*)-2-[(2*R*,3*R*,4*S*,5*R*,6*R*)-4,5-dihydroxy-6-(hydroxymethyl)-2-[(1*R*,2*S*,4*S*,5'*S*,6*R*,7*S*,8*R*,9*S*,12*S*,13*S*,16*S*,18*R*)-5',7,9,13-tetramethylspiro[5-oxapentacyclo[10.8.0.0^2,9^.0^4,8^.0^13,18^]icosane-6,2'-oxane]-16-yl]oxyoxan-3-yl]oxy-6-(hydroxymethyl)oxane-3,4,5-triol, CAS no: 41059-79-4) is a natural steroidal saponin with multiple-pharmacological activities, and it is primary isolated from Chinese Materia Medica *Anemarrhena asphodeloides* Bunge (well-known as Zhimu in Chinese) (**Figure 1**) which has been used for treatment various diseases including arthralgia, hematochezia, cough, hemoptysis, and so on, in traditional Chinese medicine (Wang et al., 2014). Phytochemistry studies have identified more than 100 compounds from *A. asphodeloides* Bunge, and the main constitutes are steroidal saponins, flavonoids, phenylpropanoids, alkaloids, steroids, organic acids, anthraquinones, and so on (Wang et al., 2014). The total saponins, which are rich in rhizome, could be extracted by hot water under reflux and purified by EtOAc, n-BuOH, and H_2_O, and the content of saponins is more than 6% (Wang et al., 2014; Yang et al., 2016; Nian et al., 2017). Timo AIII, Timosaponin BII (Timo BII) and sarsasapogenin are three main active saponins isolated from *A. asphodeloides* Bunge (**Figure 1**), and they have been identified as quality control and pharmacokinetic markers of diverse *A. asphodeloides* Bunge-contained Chinese herb formulas, such as TongGuanWan, Rhizoma Anemarrhenae-Phellodendron herb pair, guizhi-shaoyao-zhimu herb pair, zhimu-baihe herb pair, and so on (Tang et al., 2012; Wang et al., 2014; Tang et al., 2015; Yang et al., 2018). The biotransformation of Timo AIII from Timo BII could been mediated by β-D-glycosidase (Lu et al., 2016). Lu et al. also developed an enzyme associated five-step preparation method to produce high yield and purity Timo AIII from *A. asphodeloides* Bunge, which allowed us obtain efficient amount of Timo AIII for further study and product development (Lu et al., 2016). Although Timo AIII and Timo BII are mainly metabolized to sarsasapogenin *in vivo*, the sugar chain plays important roles in their pharmacological activities (Sy et al., 2008; Lee et al., 2010; Wang et al., 2010). The sugar chain in Timo AIII is indispensable to its pharmacological activities, and conversion of Timo BII to Timo AIII enhanced its cytotoxicity (King et al., 2009). So, Timo AIII presented most potential anti-cancer activity due to its specific sugar chain binding site. However, the hydrophobicity and low bioavailability of Timo AIII limited its efficacy *in vivo*, and many studies also focused on derivatization or drug delivery system design based on Timo AIII (Wang et al., 2010; Hu et al., 2011; Kim et al., 2014; Lu et al., 2018).

Timo AIII affected different numerous cellular signaling pathways and presented efficacy in different cell types and various disease models both *in vitro* and *in vivo*, such as cancer, Alzheimer's disease, depression, diabetic mellitus, colitis, and so on. Timo AIII dramatically inhibited the cancer cell growth at micromole level of concentration and selectively reduced the cancer cell viability but not normal cell (King et al., 2009). Thus, Timo AIII was proposed as a potent anti-cancer agent, and its anti-cancer activity and underlying mechanisms were most investigated in the previous studies (Han et al., 2018). In this review, we comprehensively summarized the pharmacological activity, pharmacokinetics, and toxicity of Timo AIII according to the literatures in the recent decades. And this review provides an overview of the previous research related to Timo AIII, which is benefit for its further study and drug development.

## Pharmacological Activities of Timo AIII

### Cancer

#### Cytotoxicity

Timo AIII and Timo BII are two well-known steroidal saponins in *Anemarrhena asphodeloides* Bunge while Timo BII presented less cytotoxic effect than Timo AIII in cancer cells (King et al., 2009). Induction of tumor cell death by cytotoxicity agent is the important mechanism of current cancer chemotherapy (Wang et al., 2013). Selectively kill cancer cell, regardless of normal cell, is the basic principle in cytotoxic anti-cancer drug development. Timo AIII could cause cell death in cancer cell but not in normal cell in the certain concentration (King et al., 2009; Wang et al., 2013; Zhou et al., 2020). In addition, previous studies indicated that Timo AIII presented cytotoxicity effects in various kinds of cancer cells including breast cancer, hepatocellular cancer, cervical cancer, colon cancer, nasopharyngeal cancer, pancreatic cancer, lung cancer, renal cancer, chronic myelogenous leukemia, ovarian carcinoma, osteosarcoma, leukemia, melanoma, and so on. The detection methods and corresponding concentrations which presented cytotoxicity effect and/or IC50 in cancer and normal cell lines were summarized in **Tables 1** and **2**. And the anti-cancer effects and underlying mechanisms of Timo AIII were most studied in breast cancer and hepatocellular cancer. However, only a small part of studies employed a positive control when evaluating the cytotoxicity of Timo AIII in their studies (**Tables 1** and **2**). These results also indicated that the cytotoxic effect of Timo AIII to both cancer and normal cells were not all the same in the different contexts. In addition, the dosage of Timo AIII examined in animal models including mice, rat, and zebrafish were listed in **Table 3**.

**Table 1:** The cytotoxicity effects of Timo AIII in different cancer cell lines.

Cell line	Species	Category	Detection method	Tested concentration or IC50	Positive control	Reference
**BT474**	Human	Breast cancer	Annexin-PI staining	1~10 µM (24 h) and IC50 < 2.5µM(24 h)	None	(Kang et al., 2011)
**MDA-MB-231**	Human	Breast cancer	Annexin-PI staining	1~10 µM (24 h)	None	(Kang et al., 2011)
			CCK-8 assay	6–20 µM (24 h)	None	(Nho et al., 2016)
			MTT assay	0.001–1 µM (48 h)	None	(Tsai et al., 2013)
			MTT assay	2–4 µM and IC50, 2 µM (48 h)	None	(Gergely et al., 2018)
**MCF10A**	Human	Breast cancer	Annexin-PI staining	1–10 µM (24 h)	None	(King et al., 2009)
**MCF-7**	Human	Breast cancer	MTT assay	1–100 µM and IC50=~ 10 µM (48 h)	None	(Sy et al., 2008)
			MTT assay	2–4 µM and IC50 = 2 µM (48 h)	None	(Gergely et al., 2018)
**HepG2**	Human	Hepatocellular cancer	MTT assay	1–100 µM and IC50=~ 10 µM (48 h)	None	(Sy et al., 2008)
			MTT assay	3.125–50 µM (24 and 48 h)	None	(Wang et al., 2013)
			CCK-8 assay	6–20 µM (6, 24, and 48 h) and IC50 = 15.41 µM (24 h)	None	(Nho et al., 2016)
**Hep3B**	Human	Hepatocellular cancer	MTT assay	3.125~50 µM (24 and 48 h)	None	(Wang et al., 2013)
**PLC/PRF/5**	Human	Hepatocellular cancer	MTT assay	3.125~50 µM (24 and 48 h)	None	(Wang et al., 2013)
**MHCC97L**	Human	Hepatocellular cancer	MTT assay	3.125~50 µM (24 and 48 h)	None	(Wang et al., 2013)
**HeLa**	Human	Cervical cancer	MTT assay	1–100 µM and IC50=~ 10 µM (48 h)	Sarsasapogenin (50 µM)	(Sy et al., 2008)
**SUNE-1**	Human	Nasopharyngeal cancer	MTT assay	1–100 µM and IC50=~ 10 µM (48 h)	None	(Sy et al., 2008)
**HCT-15**	Human	Colon cancer	SRB protein staining	2.5–20 µM and IC50 = 6.1 µM (3 days)	None	(Kang et al., 2011)
**HCT-116**	Human	Colon cancer	SRB protein staining	IC50 = 5.5 µM (3 days)	None	(Kang et al., 2011)
**HT-29**	Human	Colon cancer	SRB protein staining	IC50 = 10.1 µM (3 days)	None	(Kang et al., 2011)
**SW-480**	Human	Colon cancer	SRB protein staining	IC50 = 13.1 µM (3 days)	None	(Kang et al., 2011)
**SW-620**	Human	Colon cancer	SRB protein staining	IC50 = 11.1 µM (3 days)	None	(Kang et al., 2011)
**A549**	Human	Lung cancer	CCK-8 assay	3–30 µM (24 h) and IC50 = ~10 µM	None	(Jung et al., 2016)
			CCK-8 assay	6–20 µM (24 h)	None	(Nho et al., 2016)
**A549/T**	Human	Lung cancer	MTT assay	1.56–100 µM and IC50 = 5.12 µM (24 h)	None	(Song et al., 2019)
**A375-S2**	Human	Melanoma	MTT assay	1–8 µM (24 h)	None	(Wang et al., 2017)
**B16-F10**	Murine	Melanoma	MTS assay	10–100 nM (24 h)	None	(Kim et al., 2016)
**WM-115**	Human	Melanoma	MTS assay	10–100 nM (24 h)	None	(Kim et al., 2016)
**PANC-1**	Human	Pancreatic cancer	Modified MTT assay*	5–20 µM (24 and 48 h)	Gemcitabine (1 mM)	(MarElia et al., 2018)
**BxPC-3**	Human	Pancreatic cancer	Modified MTT assay*	5–20 µM (24 and 48 h)	Gemcitabine (1 mM)	(MarElia et al., 2018)
**AsPC-1**	Human	Pancreatic cancer	MTT assay	0–100 µM and IC50 = 22.1 µM (24 h)	Gemcitabine (5 µM)	(Kim et al., 2019b)
**786-O**	Human	Renal cancer	MTT assay	2–8 µM (24 h)	None	(Chiang et al., 2019)
**A-498**	Human	Renal cancer	MTT assay	2–8 µM (24 h)	None	(Chiang et al., 2019)
**ACHN**	Human	Renal cancer	MTT assay	2–8 µM (24 h)	None	(Chiang et al., 2019)
**K562**	Human	Chronic myelogenous leukemia	CCK-8 assay	0.2–1.6 mg/L and IC50 = 1.01 mg/L (24h)	Adriamycin (4-32µg/m)	(Chen et al., 2016)
**K562/ADM**	Human	Chronic myelogenous leukemia	CCK-8 assay	16–128 mg/L and IC50 = 32.18 mg/L (24h)	Adriamycin (4-32µg/m)	(Chen et al., 2016)
**A2780/T**	Human	Ovarian carcinoma	MTT assay	1.56–100 µM and IC50 = 4.64 µM (24 h)	None	(Song et al., 2019)
**MG63**	Human	Osteosarcoma	CCK-8 assay	3–15 µM (24 h) and IC50 = 12 µM	None	(Jung and Lee, 2019)
**U2OS**	Human	Osteosarcoma	CCK-8 assay	3–15 µM (24 h)	None	(Jung and Lee, 2019)
**Jurkat cell**	Human	T−cell acute lymphoblastic leukemia	CCK-8 assay	2–32 µM (24, 48, and 72 h)	None	(Wang et al., 2019)

SRB, sulforhodamine-B; *CellTiter 96 non-radioactive cell proliferation assay (MTT, Promega).

**Table 2:** The cytotoxicity effects of Timo AIII in various normal cells.

Cell line	Species	Category	Detection method	Tested concentration or IC50	Positive control	Reference
**MRS-5**	Human	Normal lung epithelial	SRB protein staining	IC50 > 50 µM (3 days)	None	(Kang et al., 2011)
**Hs68**	Human	Normal lung fibroblast	SRB protein staining	IC50 > 50 µM (3 days)	None	(Kang et al., 2011)
**L-02**	Human	Normal hepatocyte	MTT assay	8–512 µM and IC50 > 128 µM (24 h)	None	(Wang et al., 2013)
**Chang**	Human	Normal hepatocyte	CCK-8 assay	6–20 µM (24 h)	None	(Nho et al., 2016)
**hPBMC**	Human	Peripheral blood mononuclear cells	MTT assay	2.5–40 µM (24 h)	5-FU and paclitaxel (2.5–40 µM)	(Wang et al., 2017)
**HUVEC**	Human	Umbilical vein endothelial cell	MTT assay	0.5–8 µM (24 h)	None	(Zhou et al., 2020)
**SK-N-SH**	Human	Neuroblastoma	Western blot assay	2.5–10 µM (1 h)	Tacrine (5 µM)	(Lee et al., 2009)
**HEK**	Human	Epidermal keratinocyte	MTT assay	1–200 nM (24 h)	None	(Kim et al., 2019a)
**HDF**	Human	Dermal fibroblast	Cell migration assay	5–20 nM (12 h)	None	(Kim et al., 2019a)
**Primary macrophage**	Mice	Peritoneal macrophage	Trypan blue method	2–5 µM (20 h)	None	(Lim et al., 2015)
**BV-2**	Mice	Neuronal microglia	Western blot assay	2.5–10 µM (1 h)	Tacrine (5 µM)	(Lee et al., 2009)
**RBL-2H3**	Rat	Cancerous basophil cells	ELISA assay	20 and 50 µM (4 h)	Dexamethasone (10 µM)	(Lee et al., 2010)

SRB, sulforhodamine-B.

**Table 3:** The dose range of Timo AIII examined in animal models.

Animal	Species	Administration approach	Experimental duration	Tested dosage	Reference
**Tübingen (TU)** **(1 dpf)**	Zebrafish	Immersed in drug-contained culture medium	24 h	0.5, 1, and 2 µM	(Zhou et al., 2020)
**Tg(Fli-1: EGFP)y1** **(1 dpf)**	Zebrafish	Immersed in drug-contained culture medium	12 h	0.5, 1, 2, and 3 µM	(Zhou et al., 2020)
**Nude mice** **(female)**	Mice	Intraperitoneal injection (3 times per week)	3 weeks	7.5 mg/kg	(Wang et al., 2013)
**BALB/c nude mice** **(male, 5 weeks old)**	Mice	Intraperitoneal injection (3 times per week)	30 days	2 and 5 mg/kg	(Kang et al., 2011)
**C57BL / 6**	Mice	Intraperitoneal injection	14 days	12.5 and 25 mg/kg	(Kim et al., 2016)
**BALB/c nude mice** **(male)**	Mice	Intraperitoneal injection (every two days)	10 days	2.5 and 5 mg/kg	(Song et al., 2019)
**ICR** **(male)**	Mice	Oral administration or intraperitoneal injection	1 h	5, 20, and 50 mg/kg	(Lee et al., 2010)
**BALB/c nude mice** **(male, 7 weeks old)**	Mice	Oral administration (twice daily)	5 days	10, 20, and 40 mg/kg	(Cong et al., 2016)
**Wistar** **(male, 8 weeks old)**	Rat	Oral administration (twice daily)	5 days	10, 20, and 40 mg/kg	(Cong et al., 2016)
**BALB/c nude mice** **(5–6 weeks old)**	Mice	Intraperitoneal injected (3 times per week)	30 days	7.5 mg/kg	(Lu et al., 2018)
**ICR** **(6-8 weeks old)**	Mice	Oral administration (once daily)	7 days	30, 90, and 270 mg/kg	(Han et al., 2015)
**KK-Ay**	Mice	Oral administration (once daily)	8 weeks	30, 90, and 270 mg/kg	(Han et al., 2015)
**SD** **(male)**	Rat	Oral administration	14 days	100 mg/kg	(Wu et al., 2014)
**BALB/c nude mice** **(male)**	Mice	Intraperitoneally injected (every two days)	10 days	2.5 and 5 mg/kg	(Song et al., 2019)
**ICR** **(male)**	Mice	Oral administration	1 and 5 h	10, 20, or 40 mg/kg	(Lee et al., 2009)
**SD** **(6 weeks old)**	Rat	Oral administration (once a day)	7 days	50–200 μg/kg	(Shin et al., 2008)
**ddY** **(male,4 weeks old)**	Mice	Intravenously Injected	4-5 weeks	10 mg/kg	(Kimura et al., 1996)

dpf, day post fertilization; ICR, Institute of Cancer Research.

#### Promotion of Cell Apoptosis and Cell Cycle Arrest

Induction of cell apoptosis and cell cycle arrest, which stops cancer cell proliferation and causes cell death, is the current main strategy in cancer treatment. The anti-cancer effect of Timo AIII was firstly reported ten years ago in 2008 by Sy et al. They identified that prolong treatment Timo AIII increased cytochrome c release and caspase activation in HeLa cells, and this pro-apoptosis effect of Timo AIII was mediated by over production of ROS and mitochondrial dysfunction (Sy et al., 2008). However, the anti-cancer effects of Timo AIII were most studied in breast cancer and hepatocellular cancer in the past decades. King et al. found that Timo AIII promoted breast cancer cell BT474, MDAMB231 and MCF10A apoptosis in a concentration-dependent manner, and the underlying mechanisms was co-related with target of rapamycin complex 1 (TORC1) inhibition and endoplasmic reticulum (ER) stress stimulated-apoptosis as well as the inhibition of major cell proliferation signaling transduction pathways (King et al., 2009). In the research field of hepatocellular cancer, Wang et al. found that Timo AIII enhanced the apoptosis cell population by activation of poly-ADP ribose polymerase (PARP) and caspase 3, and Z-VAD-FMK which is a cellular apoptosis inhibitor suppressed Timo AIII-induced cytotoxicity in human hepatocellular cancer cell lines including HepG2, MHCC97L, PLC/PRF/5 and Hep3B (Wang et al., 2013). And Timo AIII induced tumor cell apoptosis and increased cleavage PARP and caspase 3 expression in xenografted tumor mice model (Wang et al., 2013). In addition, Wang et al. proved that Timo AIIII reduced the expression of X-linked inhibitor of apoptosis protein (XIAP), which is one of the inhibitor of apoptosis proteins (IAP), in both hepatocellular cancer cells *in vitro* and *in vivo*, and suppression of XIAP by siRNA reduced the toxic sensitivity of Timo AIII in hepatocellular cancer cells (Wang et al., 2013). These results indicated that Timo AIII reduced the tumor growth by modulation of XIAP-mediated cellular caspase active apoptosis in hepatocellular cancer. Moreover, Kyoung et al. found that the anti-cancer effect of Timo AIII was most effective in HepG 2 liver cancer cell among MDA-MB-231 breast cancer cell, A549 no-small-cell lung cancer cell and Hep3B liver cancer cell (Nho et al., 2016). Timo AIII induced more than 90% cell apoptosis on the concentration of 15 µM, and the IC 50 (24h) was 15.41 µM in HepG2 cell. And Timo AIII-promoted cell apoptosis was associated with the decreased expression of Bcl-2, Mcl-1 and IAP family, and the increased release of cytochrome c and activities of caspase family including caspase 3, 7, 8, and 9 (Nho et al., 2016).

In addition, Timo AIII also presented efficacy in colon cancer, pancreatic cancer, lung cancer, osteosarcoma, melanoma, and leukemia. In colon cancer, human colon cancer HCT-15 cells treated various concentrations of Timo AIII for 12 or 24 h appeared different degree of G_0_/G_1_ and G_2_/M phase cell cycle arrest, and the cell cycle regulation effect of Timo AIII was associated with the down-regulation of cyclin A, cyclin B1, CDK2, CDK4, pRb, proliferating cell nuclear antigen (PCNA) and c-Myc (Kang et al., 2011). Moreover, Timo AIII promoted cell apoptosis by induction of DNA fragmentation, activation of caspases, induction of cleaved-PARP and reduction of Bcl-xL and Bcl-2 expression in HCT-15 cells *in vitro*. In line with this results, Timo AIII significantly decreased the tumor volume in HCT-15 cell-bearing athymic nude mice *in vivo* (Kang et al., 2011). In melanoma, Timo AIII arrested the cell cycle at G_0_/G_1_ phase and enhanced the cell apoptosis by up-regulation of cleavage-caspase 3 expression in human melanoma A375-S2 cells (Wang et al., 2017). And Timo AIII enhanced the expression of iNOS which resulted in the elevated release of NO, and Timo AIII-induced cleavage of caspase 3 was attenuated by iNOS inhibitors DTT and 1400W in A375-S2 cells. Timo AIII increased the expression of JNK and ERK, and their inhibitors enhanced Timo AIII-induced cell death but decreased cleavage-caspase 3 expression in A375-S2 cells (Wang et al., 2017). Thus, JNK and ERK seem play protective roles in the Timo AIII-induced cell death. In pancreatic cancer, Kim et al. demonstrated that Timo AIII increased the number of apoptotic cells which accompanied with the reduction of mRNA expression of anti-apoptosis proteins survivin, Bcl-2 and Bcl-xl, and Timo AIII elevated cell cycle distribution at sub-G1 phase which might result from the down-regulation of cell cycle regulators cyclin-D and upregulation of cyclin-dependent inhibitor p21 in human pancreatic cancer cell AsPC-1 (Kim et al., 2019b). And combination commercial available anti-cancer drug dasatinib, which is a Src inhibitor, with Timo AIII enhanced the pro-apoptosis effect by regulation of PARP and caspase 3 expressions in AsPC-1 cells (Kim et al., 2019b). Interestingly, Timo AIII enhanced the ERK/Src phosphorylation at low concentration while decreased both the amount of phosphorylated and total protein at high concentration (Kim et al., 2019b), which revealed that high concentration of Timo AIII mainly resulted in cell death. Timo AIII also inhibited the tumor growth by activation of caspase-3 which stimulated cellular apoptosis on pancreatic cancer PANC-1 cell-xenograft nude mice model (Pan et al., 2013). In osteosarcoma and lung cancer, Timo AIII significantly induced cell apoptosis by regulation of caspase 3, caspase 7 and PARP expressions in MG63 human osteosarcoma cells (Jung and Lee, 2019), and induced cell apoptosis and cell cycle arrest at G_0_/G_1_ phase in human no-small-cell lung cancer cell A549 (Jung et al., 2016). Timo AIII also concentration-dependently induced cell apoptosis by upregulation of Bax and down regulation of Bcl-2 in T−cell acute lymphoblastic leukemia Jurkat cells (Wang et al., 2019).

**Figure 2:**
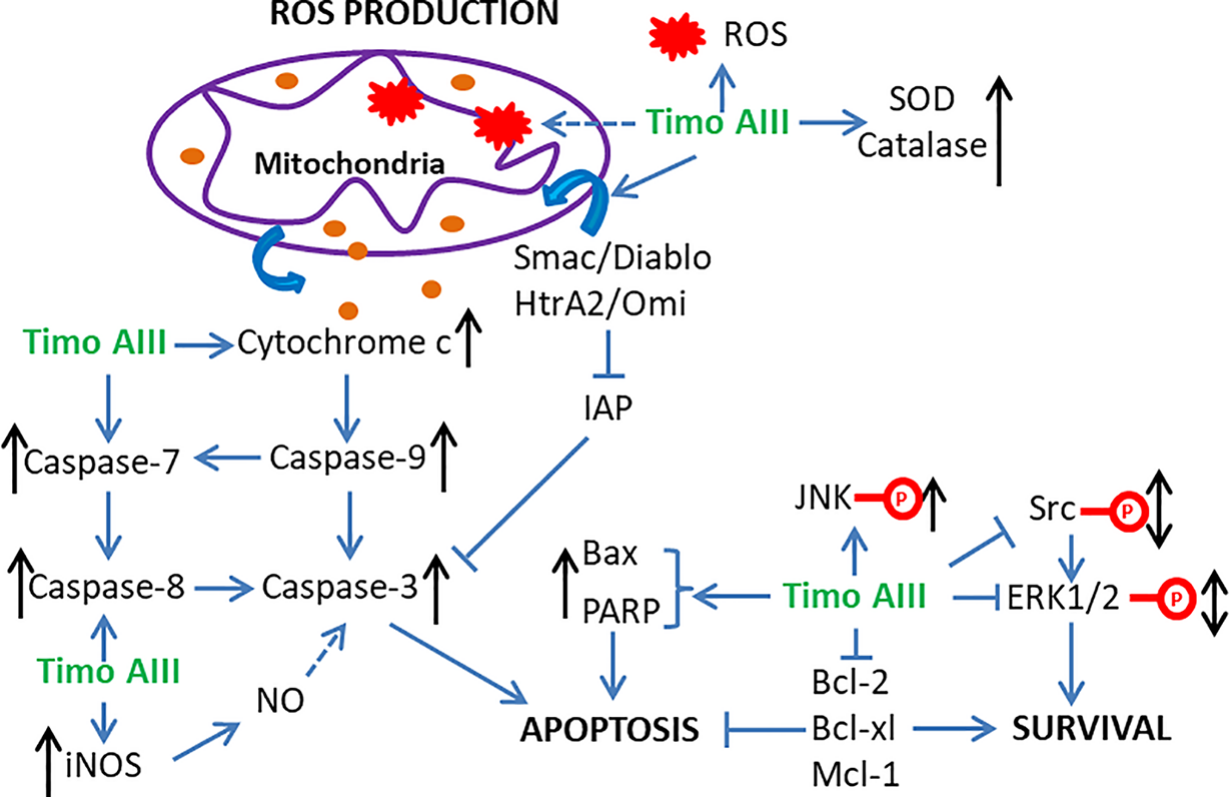
Schematic overview of the underlying mechanisms related to the induction of apoptosis, ROS production, and mitochondrial dysfunction by Timo AIII.

**Figure 3:**
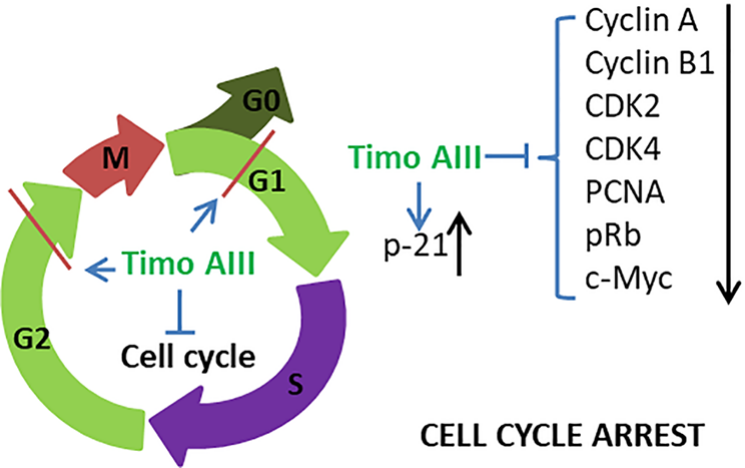
Schematic overview of the underlying mechanisms related to the cell cycle arrest effect of Timo AIII.

Thus, we could conclude that the anti-cancer efficacy of Timo AIII was well studied, especially hepatocellular cancer and breast cancer, and the mainly underlying mechanism was induction of cell apoptosis (**Figure 2**) and cell cycle arrest (**Figure 3**). In addition, the XIAP might be potential pharmacological target of Timo AIII.

#### Induction of ER Stress, ROS Generation, and Mitochondrial Dysfunction

**Figure 4:**
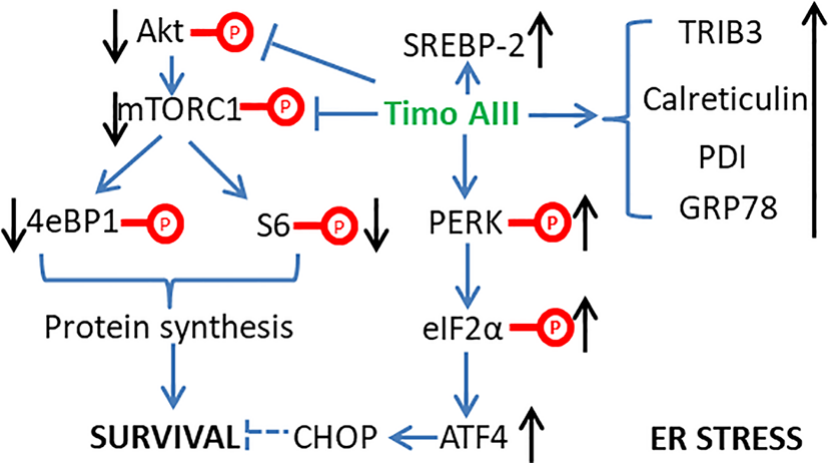
Schematic overview of the underlying mechanisms related to the induction of ER stress by Timo AIII.

Normal endoplasmic reticulum (ER) function is essential for maintaining the cellular homeostasis, and ER stress presents in cancer cells due to its nutrient limitative and hypoxic tumor micro-environment (Cubillos-Ruiz et al., 2017). However, chronic ER stress stimulated activation of cellular apoptosis pathways which could result in cancer cell death, and the anti-cancer effects of various natural products were resulted from the induction of intracellular ER stress (Kim and Kim, 2018). King et al. found that Timo AIII time-dependently enhanced the ER stress by up-regulating the protein levels of PDI, calreticulin, GRP78, ATF4, and TRIB3 and the phosphorylation levels of eIF2α and PERK, which are ER stress markers (King et al., 2009). And Timo AIII also enhanced the genes expression in cholesterol biosynthesis pathways in breast cancer cell BT474, MDAMB231 and MCF10A. However, the total cholesterol levels were not changed and the cholesterol biosynthesis regulator SREBP-2 was significantly activated in Timo AIII treated cell (King et al., 2009). Thus, the cell death induced by Timo AIII was not due to the biosynthesis of cholesterol, and the regulation of cholesterol biosynthesis by Timo AIII supported its ER stress induction activity (**Figure 4**).

Mitochondria regulates many cellular physical functions including energy metabolism, reactive oxidant species (ROS) generation and apoptosis, and Chiu et al. claimed that mitochondria played a central role in cancer development and suggested that mitochondria-targeted therapeutic approach was valuable in cancer (Chiu et al., 2020). Kyoung et al. found that Timo AIII dramatically induced apoptosis in HepG2 cells, which might be co-related to the translocation of Smac/Diablo and HtrA2/Omi from cytosol to mitochondria consequently triggered cell apoptosis (Nho et al., 2016). ROS could be either cause or result of mitochondria dysfunction which promotes disruption of intracellular homeostasis and cancer cell death (Kudryavtseva et al., 2016; Park et al., 2019; Zhou J. et al., 2019). Timo AIII induced intracellular ROS accumulation which could be cleared by anti-oxidant NAC and might be an important cause of cell death in triple negative breast cancer cell MDA-MB-231 (Tsai et al., 2013). Consistently, Timo AIII concentration-dependently increased the intracellular ROS and anti-oxidant enzymes including SOD and catalase in HeLa cells, which indicated that Timo AIII impaired intracellular redox homeostasis (Sy et al., 2008). In addition, Timo AIII destroyed mitochondrial membrane potential, mitochondrial permeability transition, and release of cytochrome c, and the mitochondria was the main sources of ROS-induced by Timo AIII in HeLa cells (Sy et al., 2008). So, targeting ROS production and mitochondria dysfunction might be essential mechanisms for Timo AIII-caused cancer cell death (**Figure 2**).

#### Anti-Angiogenesis

**Figure 5:**
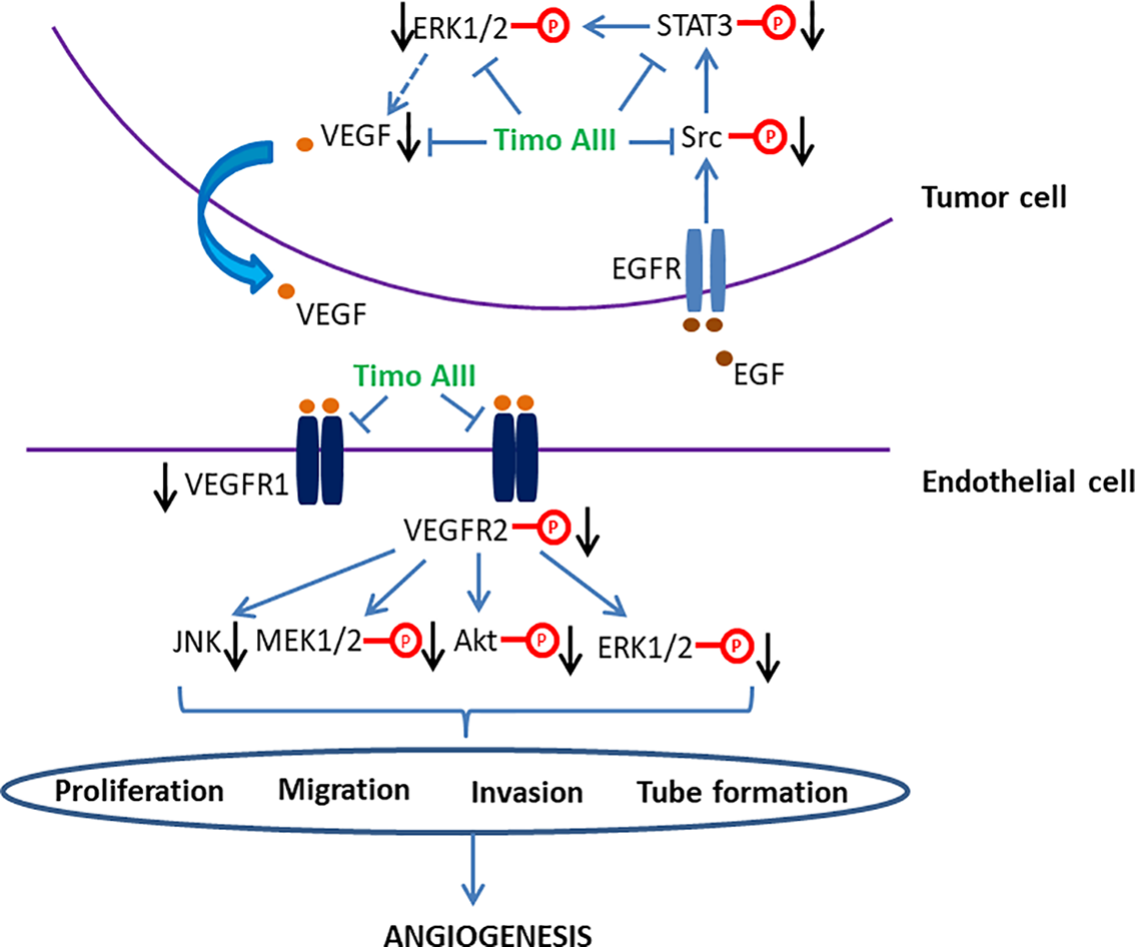
Schematic overview of the signaling pathways related to the anti-angiogenesis activity of Timo AIII.

Tumor angiogenesis, which is triggered by tumor-secreted angiogenic factors, transports nutrient to tumor and promotes tumor growth and metastasis. Targeting tumor angiogenesis and vascular normalization have been a promising anti-cancer drug development strategy, and several agents have been developed (Viallard and Larrivee, 2017; Jaszai and Schmidt, 2019). The anti-tumor effect of Timo AIII on pancreatic cancer cell PANC-1 bearing nude mice model was co-related to the down-regulation of mRNA and protein levels of the angiogenic factor VEGF (Pan et al., 2013). Timo AIII inhibited the mRNA expression of VEGF-1, and suppressed the EGF-triggered Src/STAT3/ERK signaling pathway activation in a concentration-dependent manner in pancreatic cancer cell AsPC-1 (Kim et al., 2019b). Zebrafish and vascular endothelial cell have been considered as the well-known *in vivo* and *in vitro* models for anti-angiogenesis activity evaluation with the advantages of low cost, time saving, and easy observation (Norrby, 2006; Delvecchio et al., 2011). Zhou et al. found that Timo AIII presented anti-angiogenesis effect in zebrafish *in vivo* and human umbilical vein endothelial cells (HUVECs) *in vitro* (Zhou et al., 2020). Timo AIII inhibited the intersegmental vessels (ISVs) and sub-intestinal vessels (SIVs) growth in transgenic zebrafish line Tg(Fli-1: EGFP)^y1^ which expressed enhanced green fluorescence protein (EGFP) in vascular endothelial cells. Timo AIII also inhibited the endothelial cell proliferation, migration, invasion, and tube formation in HUVECs. And the underlying mechanism might be closely related to the down-regulation of VEGFRs and suppression of VEGF/PI3K/Akt/MAPK signaling pathway. So, the anti-tumor angiogenesis effect of Timo AIII also contributes to its anti-cancer capability, and VEGFR might be potential pharmacological target of Timo AIII (**Figure 5**).

#### Anti-Metastasis

Most of the cancer patients die from cancer metastasis which consists of multiple physical processes and have long been associated with cancer cell migration and invasion (Guan, 2015). Matrix metalloproteinase (MMP) is the vital pharmacological target in cancer progression, which degrades extracellular matrix, affects the cancer microenvironment, and initiates cancer metastasis (Shay et al., 2015; Lyu et al., 2019). Various signaling pathways, including MAPKs, src/FAK, beta-catenin, NF-κB, STAT3, and so on are involved in the regulation of MMPs expressions (Pan et al., 2011; Merchant et al., 2017). Timo AIII significantly inhibited the proteolytic activity and mRNA expression of MMP-2/9 which are key regulators in cell migration and invasion in human no-small-cell lung cancer cells A549 and H1299, and the underlying mechanisms might be related to the suppression of ERK1/2, Src/FAK, and beta-catenin signaling pathways (Jung et al., 2016). Consistently, Timo AIII significantly decreased MMP-2/9 expression, and inhibited cell migration and invasion in human osteosarcoma cells MG63 and U2OS. The underlying mechanism was involved in suppression of Src/FAK/MAPKs signaling pathway and down-regulation of transcription factors CREB and b-catenin (Jung and Lee, 2019). In addition, Timo AIII decreased mRNA expression of MMP-9 in human pancreatic cancer cell AsPC-1 (Kim et al., 2019b) and suppressed HGF-induced MMP-9 expression in triple negative breast cancer cell MDA-MB-231 (Tsai et al., 2013).

MicroRNAs (miRNAs), which are small-noncoding RNA molecules and regulate the target genes expression, are classified as oncomiRs (tumor inducers) and tumor suppressor miRNA. And miRNA were well recognized for cancer treatment and diagnose in recent decades (Katz et al., 2015; Qadir and Faheem, 2017; Daoud et al., 2019). The expression level of cancer stem cell phenotype regulator B-cell-specific Moloney murine leukemia virus integration site 1 (BMI1), which is also a component of the polycomb repressive complex 1 (PRC1) and its transcription is dramatically regulated by Myc, is up-regulated in various cancers especially breast cancers (Dimri et al., 2015; Hiraki et al., 2017). Dimiri et al. suggested that the oncogenic activity of BMI1 was also mediated by miR-200c/141 cluster-BMI1 auto-regulatory loop in cancer cells (Dimri et al., 2016). Timo AIII concentration-dependently induced tumor cell senescence and inhibited cell oncogenic phenotypes, including migration, invasion, and colonies formation in soft-agar in both triple negative cancer cell MDA-MB-231 and non-triple negative cancer cell MCF-7 (Gergely et al., 2018). Gergely et al. proved that the inhibitory effect of Timo AIII on cell oncogenic phenotype was mediated by the up-regulation of miR-200c/141 cluster and down-regulation of BMI1, mono-ubiquitination of histone H2A at lysine 119 (H2AUb) and Myc in both MDA-MB-231 and MCF-7 cells (Gergely et al., 2018). There are also accumulated evidences which revealed the tumor suppressive role of miR-129-5p in various cancers (Liu et al., 2019; Qiu et al., 2019; Shi et al., 2019). Timo AIII inhibited cell migration and invasion among the concentration range of 2 to 6 µM in which Timo AIII not affected the cell viability and cell cycle distribution in human renal cancer cells 786-O and A-498 (Chiang et al., 2019). And this non-toxic inhibitory effect of Timo AIII on cell migration and invasion was co-related with the down-regulation of cysteine protease cathepsin c expression, which was regulated by PI3K/Akt/miR-129-5p signaling axis (Chiang et al., 2019).

The inflammatory infiltration state of cancer microenvironment plays crucial role in cancer growth, metastasis, and resistance, which is mainly regulated by COX2/PGE2/EP and NF-κB signaling cascades (Hsu et al., 2017; Tong et al., 2018). The inhibition of these processes presented potential therapeutic effect in various cancers (Yun et al., 2016; Liu et al., 2018). Timo AIII suppressed the migration capability of murine melanoma cell B16-F10 and human melanoma cell WM-115, which were induced or not induced by COX-2 stimulator 12-O-tetradecanoylphorbal-13-acetate (TPA), and it was closely associated with its concentration-dependent inhibitory effect on endogenous COX-2 expression and PGE2 production (Kim et al., 2016). Timo AIII also reduced protein levels of NF-κB, IKKα, IκBα, and PGE2 receptors including EP2 and EP4 in B16-F10 cells (Kim et al., 2016). In line with these *in vitro* experiment results, Timo AIII significantly inhibited the metastasis of B16-F10 cells to lung with reduction of COX2 and NF-κB expression in mice *in vivo* (Kim et al., 2016). Many growth factors induce cancer cell proliferation, migration, and invasion, which contribute to cancer cell motility and intravasation (Guan, 2015). Hepatocyte growth factor (HGF), which promotes various cancer cell metastasis, triggered cancer cell migration and invasion *via* cMet-ERK-COX2 and MAPK signaling pathways (Siegfried et al., 2007; Kuang et al., 2017). Timo AIII concentration-dependently inhibited HGF-induced cell migration and invasion which were blocked by COX2 inhibitor NS398 and ERK inhibitor PD98059 in triple negative breast cancer cell MDA-MB-231 (Tsai et al., 2013). Timo AIII also significantly attenuated HGF-induced intracellular p-cMet and COX2 expressions as well as nuclear ERK phosphorylation and ATF2 expression in MDA-MBA-231 cells (Tsai et al., 2013).

**Figure 6:**
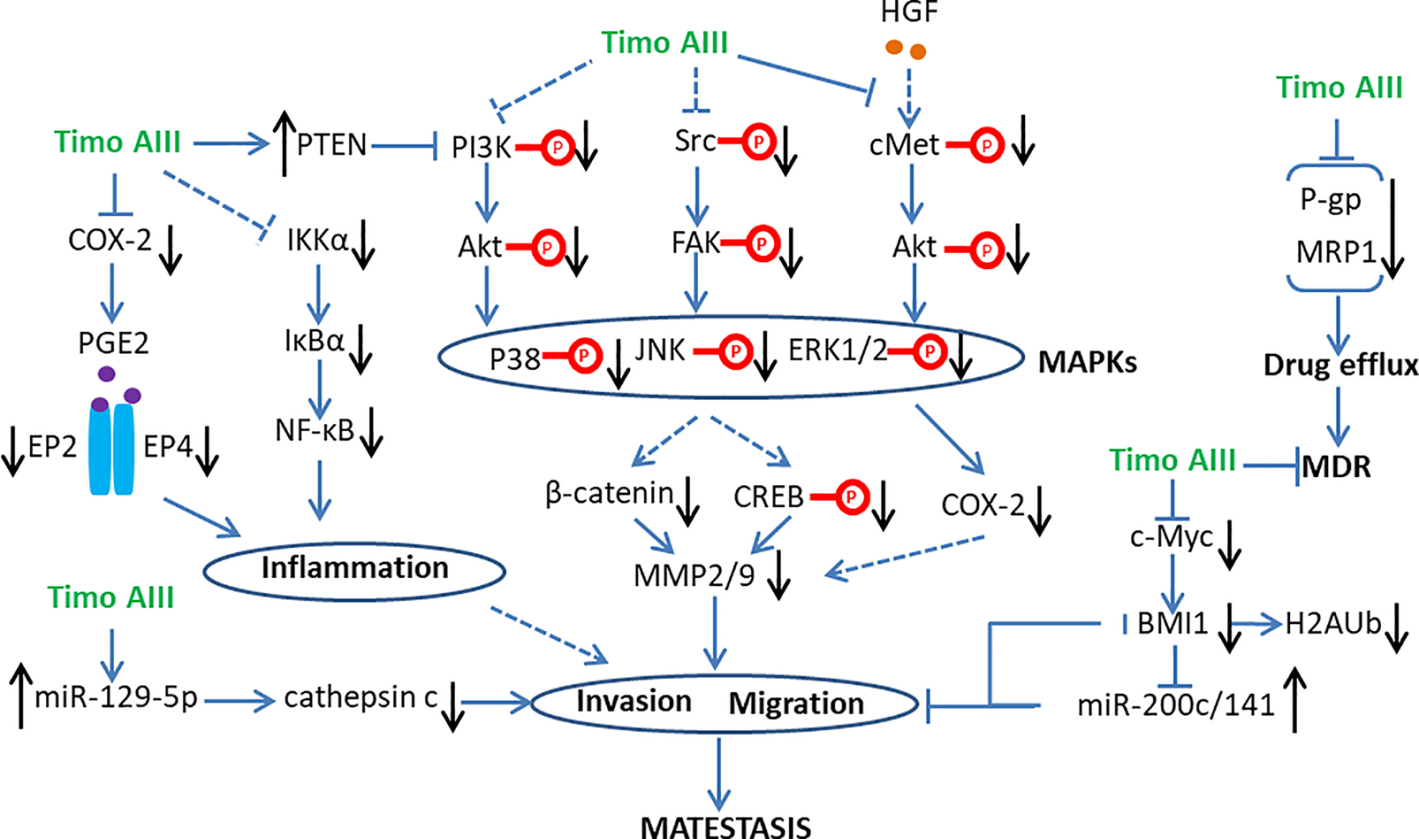
Schematic overview of the signaling pathways related to the anti-metastasis and anti-multi drug resistance (MDR) activity of Timo AIII.

Thus, Timo AIII inhibited cancer stem cell phenotype, including cell migration and invasion, and subsequently suppressed cancer metastasis by targeting MMPs, BMI1, and cancer inflammatory infiltration (**Figure 6**).

#### Anti-Resistance and Synergistic Anti-Cancer Effect

Multiple drug resistance (MDR) is a severe problem in current cancer therapy (Gottesman, 2002; Vasan et al., 2019). Elevating the sensitivity of therapeutic drug to resistant-cancer cell by combination with other agents might be a promising therapeutic strategy. Timo AIII concentration-dependently caused cytotoxicity, cell cycle arrest, and caspase-dependent apoptosis in PANC-1 and BxPC-3 cells, which were resistance to gemcitabine in different degree, and enhanced the sensitivity to gemcitabine in these pancreatic cancer cells (MarElia et al., 2018). The underlying mechanism of Timo AIII enhanced the sensitivity of gemcitabine on cell cycle dysfunction, and apoptosis was involved in activation of pro-apoptotic proteins in PI3K/Akt signaling cascade (MarElia et al., 2018). Timo AIII also inhibited PI3K/Akt signaling pathway on adriamycin (ADM)-resistant human chronic myelogenous leukemia cell K562 (Chen et al., 2016). And the anti-tumor effects of Timo AIII on taxol-resistance lung and ovarian cancers were co-related to the suppression of PI3K/AKT/mTOR and Ras/Raf/MEK/ERK signaling pathways (Song et al., 2019). In addition, Ginsenosides including compound K, Rb1, and Rc enhanced the cytotoxicity of Timo AIII in MG63 human osteosarcoma cells, in which Rb1 and Rc increased the population of apoptosis cell caused by Timo AIII (Jung and Lee, 2019). Thesis results indicate that Timo AIII could enhance the cancer cell apoptosis and suppressed the cell survival signaling, and ginsenoside might present synergistic effect on Timo AIII-induced cancer cell death and metastasis.

The metabolism and transport of intracellular drug to extra cell contributes to MDR, which is also called drug efflux. The ATP-binding cassette (ABC) transporter family including multi-drug resistance protein 1 (MDR1; also known as P-glycoprotein and ABCB1), MDR-associated protein 1 (MRP1; also known as ABCC1) and breast cancer resistance protein (BCRP; also known as ABCG2) regulates the process of drug efflux, which also overexpresses in many drug-resistant cancer cells (Holohan et al., 2013). Timo AIII increased intracellular adriamycin (ADM) concentration and presented reversal effect on ADM-resistant human chronic myelogenous leukemia cell K562 in a concentration-dependent manner, and the underlying mechanisms were related to the down-regulation of P-glycoprotein (P-gp) and MRP1 (Chen et al., 2016). Moreover, Timo AIII attenuated the expression of P-gp and enhanced cell apoptosis in taxol-resistance human lung cancer cell A549/T and human ovarian cancer A2780/T *in vitro* and A549/T cell injected nude mice *in vivo* (Song et al., 2019). Thus, the inhibition of drug efflux by down-regulation of drug transporters expression was a key mechanism account for the anti-resistant capability of Timo AIII in cancer cells (**Figure 6**).

#### Negative Regulated Cell Death by Autophagy

**Figure 7:**
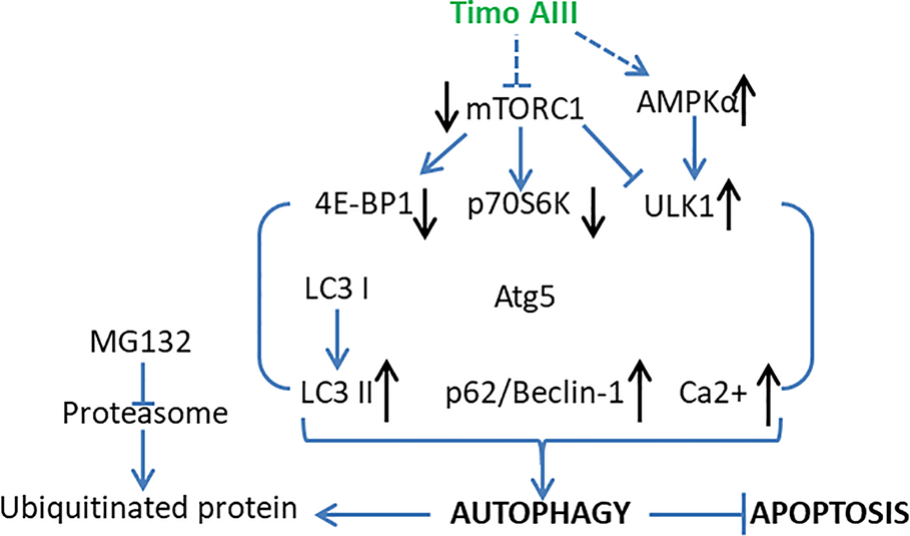
Schematic overview of the underlying mechanisms and signaling pathways related to the cellular protective autophagy activity of Timo AIII.

Autophagy characterized by the formulation of autophagic vacuoles which degrade and recycle the dysfunctional proteins and damaged organelles subsequently maintains the intracellular homeostasis (Antunes et al., 2018). Autophagy is a double-edged sword in cancer development, progression, and treatment, which could be tumor-suppressive, tumor-promoting, or neutral under different conditions (Amaravadi et al., 2016). PI3K/Akt/mTOR signaling pathway plays a key role in regulation of autophagy (Colhado Rodrigues et al., 2019). Timo AIII notably induced cellular autophagic morphology *via* inhibition of PI3K/Akt/mTOR signaling pathway in Jurkat cells (Wang et al., 2019). Lok et al. found that Timo AIII suppressed the mTOR signaling and trigged autophagy while this process was not all the same with rapamycin-induced autophagy in HeLa cells (Lok et al., 2011). Timo AIII modulated specific transcriptional mechanisms and caused the up-regulation of cholesterol biosynthesis pathways for supporting the formation of autophagic vacuoles which capture ubiquitin proteins (Lok et al., 2011). Timo AIII-induced autophagy was co-related with elevated intracellular calcium concentration and accelerated the clearance of ubiquitin protein aggregation which induced by proteinase inhibitor MG132 (Lok et al., 2011). In addition, Timo AIII inhibited the mTORC1 and promoted selective protective autophagy in breast cancer cells (King et al., 2009). The inhibition of autophagy by chloroquine enhanced the cytotoxicity of Timo AIII in MDA-MB-231 and MCF 10A cells regardless of BT474 cell (King et al., 2009). In line with this result, Timo AIII increased the accumulation and conversion of cytosol LC3 I to autophagosome membrane located LC3 II, which revealed the autophagy induction effect in HeLa cells (Lok et al., 2011), and Timo AIII-induced apoptosis was potentiated in the presence of autophagy inhibitor 3-methyladenine (3-MA) or silenced beclin-1 gene which is a key regulator of autophagy (King et al., 2009). Moreover, Timo AIII induced autophagy in hepatocellular cancer cells both *in vitro* and *in vivo*, which mediated by AMPKα activation and mTOR inhibition (Wang et al., 2013). The inhibition of autophagy by Atg5 silence increased the cytotoxicity of Timo AIII in hepatocellular cancer cells, which revealed the cyto-protective role of autophagy in Timo AIII-induced hepatocellular cancer cell death (Wang et al., 2013). Timo AIII increased autophagy vacuoles, Beclin-1 and LC3 levels in human melanoma A375-S2 cells, and Timo AIII-induced apoptosis was accelerated by the autophagy inhibitor 3-MA (Wang et al., 2017). These results indicated that Timo AIII-induced autophagy might act as the role to promote tumor cell survival *in vitro*, and mTORC1 might be the potential target of Timo AIII (**Figure 7**).

### Neuronal Disorders

#### Alzheimer**'**s Disease

**Figure 8:**
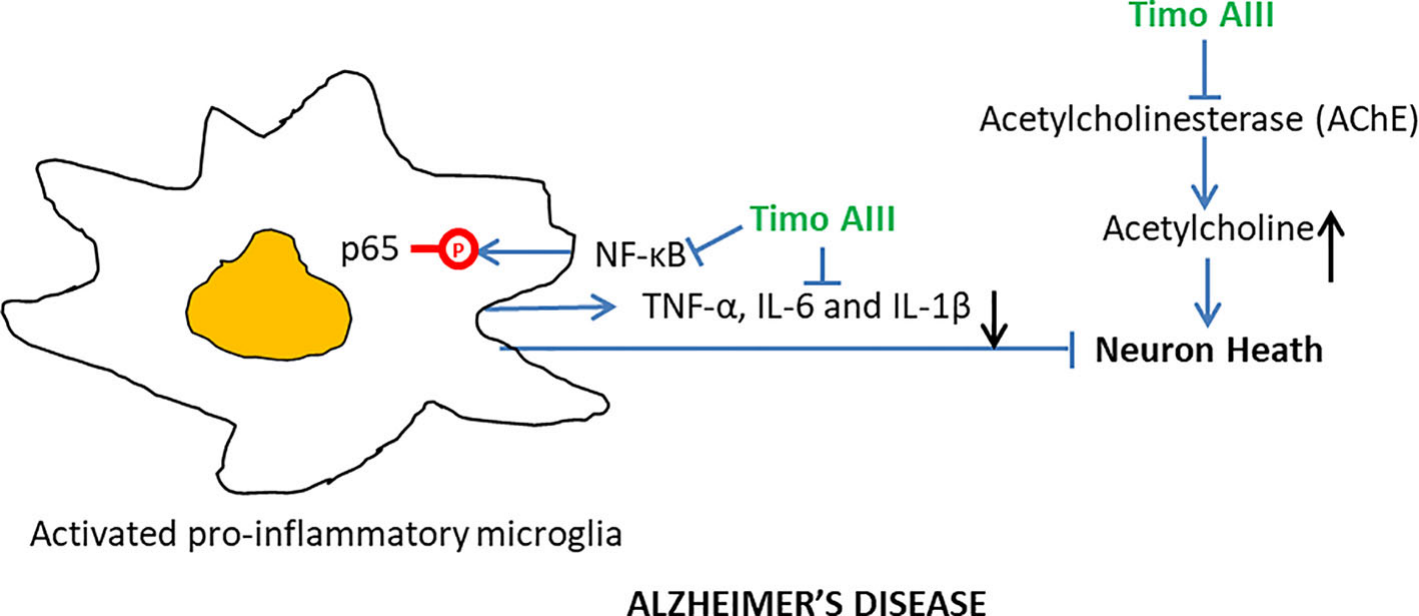
Overview of the underlying mechanisms of the Alzheimer's disease protective effect of Timo AIII.

Alzheimer's disease (AD), which is a progressive neurodegeneration disease and characterized by disorder of memory and cognitive functions, is the most common type of dementia with poor therapeutic efficacy until now (Oboudiyat et al., 2013). Several pathology features, including neuro-inflammation, accumulation of β-amyloid plaques and tau protein, and deficient cholinergic transmission were found in the brain of AD patients (Tundis et al., 2016). Acetylcholinesterase (AChE) degrades acetylcholine and lowers cholinergic activity and its selective inhibitor donepezil has been used for AD treatment in clinics (Arvanitakis et al., 2019). Lee et al. found that orally administration of Timo AIII (10, 20 and 40 mg/kg) significantly ameliorated the scopolamine-caused mice memory impairment in both passive avoidance test and Morris water maze test, and tacrine (10 mg/kg)-treated mice served as the positive control (Lee et al., 2009). Timo AIII elevated the acetylcholine level in scopolamine-treated mice brain which was correlated to its concentration dependently inhibition of the AChE activity (IC 50 = 35.4 µM). In addition, the elevation levels of pro-inflammatory cytokines including TNF-α, IL-6 and IL-1β have been found in the AD brain, and microglia triggered neuro-inflammation has been considered contribution to AD progression (Webers et al., 2019). Timo AIII decreased the expression of pro-inflammatory cytokines TNF-α and IL-1β in the brain of scopolamine-treated mice, and the underlying mechanism was associated with the suppression of NF-κB signaling pathway activation in both microglia and neuron (Lee et al., 2009). Though the anti-AD activity of Timo AIII was demonstrated *in vivo*, whether Timo AIII could cross blood-brain barrier need to be further studied. Thus, we could conclude that Timo AIII was potentially for AD therapy due to its inhibition on AChE activity and NF-κB-mediated neuro-inflammation (**Figure 8**).

#### Depression

Depression is a serious psychiatric disease and dramatically disrupted the quality of life (Smith, 2014). Although these are multiple pharmacological and physical strategies for depression treatment in clinic, various side effects accompany with the positive outcomes (Tomlinson et al., 2019). Timosaponin B-III (Timo B-III) which is also one of the main natural steroidal saponins in *A. asphodeloides* Bunge prevented from depression in a mouse postpartum depression model (Zhang et al., 2017). Timo AIII (30 mg/kg) also presented anti-depression activity according to the experiment results from open field test, tail suspension test and forced swimming test in mice, and fluoxetine-treated mice served as the positive control. However, the anti-depression effect of Timo B-III was more effective than Timo AIII (Jiang et al., 2014). Thus, further studies about the anti-depression effect of Timo B-III are more valuable than Timo AIII.

### Diabetic Mellitus

*Anemarrhena asphodeloides* Bunge is an important constitute of herb medicine formulas, like TongGuanWan, Bai-Hu-Jia-Rensheng-Tang, Rhizoma Anemarrhenae-Phellodendron herb pair and guizhi-shaoyao–*A. asphodeloides* herb pair, which have been applied for treatment of diabetic mellitus for thousands of years in China (Kimura et al., 1996; Shin et al., 2008; Tang et al., 2012; Han et al., 2015; Zhao et al., 2015). But the active constitutes of *A. asphodeloides* Bunge for diabetic mellitus treatment was not fully elucidated. The diabetic mellitus protective effect of Timo AIII was firstly investigated on streptozocin (STZ)-induced diabetic and normal mice in 1996 (Kimura et al., 1996). In diabetic mice model, Timo AIII (1 and 10 mg/kg) significantly promoted the salivary flow. And combination of CaCl_2_ (2 and 4 mg/kg) with Timo AIII (0.1 mg/kg) enhanced the pilocarpine-induced saliva secretion when compared with Timo AIII alone (Kimura et al., 1996). However, Nian et al. found that the glucosidase inhibitory effect of flavones, including mangiferin and isomangiferin, were more effective than steroidal saponins including Timo AIII and Timo BII, which revealed the more potent anti-diabetic mellitus effects of flavones than saponins isolated from *A. asphodeloides* Bunge (Nian et al., 2017). And Yuan et al. found Timo BII prevent from diabetic nephropathy by suppression the inflammation in alloxan-induced mice (Yuan et al., 2015). These results revealed that the anti-diabetic mechanisms were different between steroidal saponins and flavones ingredients of *A. asphodeloides* Bunge and the anti-diabetic mellitus activity of Timo AIII might be due to its anti-inflammatory property.

### Others

#### Anti-Platelet and Anti-Thrombotic Activity

Platelet activation and aggregation have been considered as the main pathology of many cardiovascular diseases, such as coronary heart disease, atherosclerosis, and strokes (Yeung et al., 2018). The inhibition of thromboxane prostaglandin (TP) receptor which blocked thromboxane (Tx) A2 pathway has been suggested as the efficient drug target for inhibition of platelet activation and aggregation (Fu et al., 2017; Kashiwagi et al., 2019). Timo AIII inhibited U46619-induced platelet aggregation by reduction of ADP secretion *in vitro* and prevented thrombus formation in mice *in vivo* (Cong et al., 2016). The thromboxane (Tx) A2 receptor activity and Gq signaling pathway were suppressed by Timo AIII, and the anti-platelet aggregation activity was enhanced by combination Timo AIII with current known antiplatelet agents including PGE1 and SQ29548. So, Timo AIII might be potential for development of cardiovascular drugs for its anti-platelet and anti-thrombotic activity targeting thromboxane (Tx) A2 receptor.

#### Anti-UVB Radiation

UVB radiation was harmful to skins which resulted in cellular damage, mutation, inflammation, photoaging, and consequently, photocarcinogenesis progression (Lan, 2019). Natural herbs were precious library for discovery of UV protective agents (Li et al., 2019; Mohan and Gupta, 2019). Kim et al. found that Timo AIII, which isolated from Chinese herb *A. asphodeloides* Bunge, protected against UVB-induced cell migration and invasion in both human epidermal keratinocytes (HEKs) and dermal fibroblasts (HDF), and the underlying mechanism were involved in the inhibition of Akt/MAPK signaling pathway and down-regulation of MMP9 (Kim et al., 2019a). Timo AIII reduced UVB-induced up-regulation of COX2 and inflammatory cytokines including TNF-α and IL-6 expressions, which was associated with the down-regulation of pro-inflammatory factor NF-κB. In addition, Timo AIII reduced UVB-induced DNA damage and deposition of 8-oxo-7, 8-dihydro-2'-deoxyguanosine (8-oxo-dG), which were mediated by down regulation of cell cycle arrest–related genes PCNA and SMC1. Thus, the UBV-radiation protective effect of Timo AIII might be mainly due to its anti-inflammatory, anti-cell migration, and anti-cell invasion capabilities.

#### Anti-Colitis

**Figure 9:**
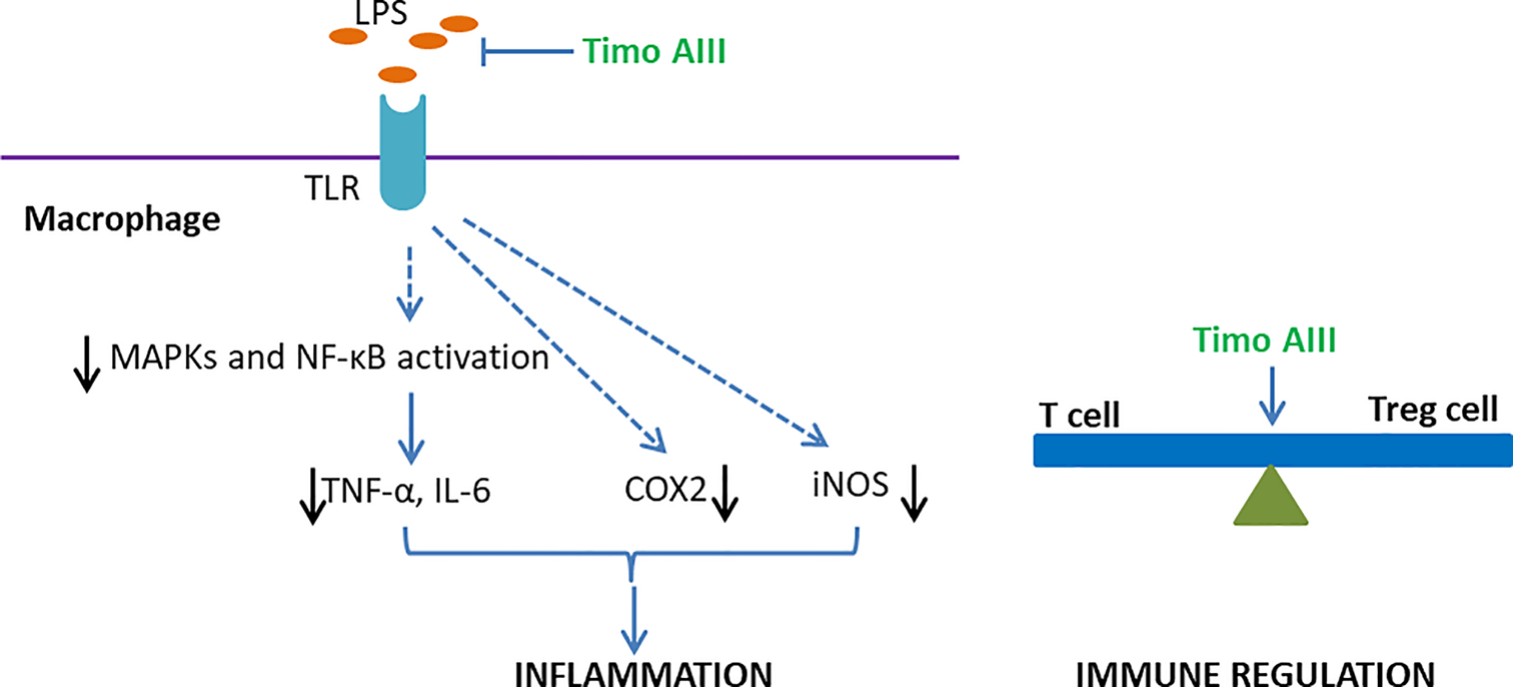
Overview of the underlying mechanisms of the anti-inflammation and immune regulation effects of Timo AIII.

Timo AIII concentration-dependently inhibited in LPS- or peptidoglycan-stimulated upregulation of COX2, iNOS, and pro-inflammatory cytokines TNF-α and IL-6 expressions in mice peritoneal macrophages, which accompanied with suppression of MAPKs and NF-κB signaling pathways activation (Lim et al., 2015). Moreover, Lim et al. found that Timo AIII reduced the amount of LPS binding to TLR and restored the balance of Th17/Treg cells in mice peritoneal macrophages, and these *in vitro* experiment results were in line with the anti-inflammatory effects of Timo AIII on 2,4,6-trinitrobenzene sulfonic acid (TNBS)-induced colitis in mice *in vivo* (**Figure 9**).

#### Anti-Allergy

Timo AIII inhibited the passive cutaneous anaphylaxis (PCA) reaction and scratching behaviors in mice while further studies revealed that sarsasapogenin, one of the metabolites of Timo AIII, was the active metabolic compound in Timo AIII-induced anti-allergy (Lee et al., 2010). Lim et al. also found that Timo AIII inhibited the differentiation of T cell to Treg cell *in vitro* (Lim et al., 2015). Thus, Timo AIII might also be able to regulate the immune system (**Figure 9**).

#### Anti-Virus

Respiratory syncytial virus (RSV) has been identified as the most important cause of lower respiratory tract infections and results in some mortality in young children and elderly (Jorquera et al., 2016; Broor et al., 2018). Although several inhibitors targeting RSV has been found over decades, there is still no antiviral drugs or vaccines approved for the prevention or treatment of RSV infections (Heylen et al., 2017). Joung et al. isolated the Timo AIII from the BuOH fraction of *A. asphodeloides* Bunge and found that Timo AIII significantly inhibited the propagation of RSV in HEp-2 cells (IC50 = 1.0 µM) (Joung et al., 2011). Furthermore, ribavirin–treated group served as the positive control with the IC50 value of 1.15 µM, which was higher than Timo AIII. Thus, we could conclude that Timo AIII presented potential anti-virus activity while its action mechanisms still needs further studies.

## Pharmacokinetics and Toxicity

Pharmacokinetics and toxicity studies play vital roles in the process of drug development. Several analytical methods were developed for detection of the saponins including Timo AIII and Timo BII in animal blood plasma after administration of single compound or herb formulas (Liu Y. et al., 2013; Sun et al., 2013; Feng et al., 2014). Timo AIII is one of the important active ingredients of Baihe Zhimu decoction which was used for depression treatment in traditional Chinese medicine for a long history in China (Yang et al., 2018). Yang et al. found that Timo AIII presented in the blood after oral administration of Baihe Zhimu decoction by an AB Sciex QTRAP ((R)) 5500 mass spectrometer in both normal and depression rats (Yang et al., 2018). Lee et al. detected the blood concentration of Timo AIII using LC-MS in mice, and they found that its maximum blood concentration was 104.7 ± 20.7 ng/µL after oral administration of Timo AIII (50 mg/kg) for 4 to 6 h (Lee et al., 2009). And this result was consistent with efficacy results that the memory-enhancing effect of Timo AIII after orally administration for 5 h was more effective than 1 h. However, Liu et al. found that the Cmax, Tmax, t1/2, and MRT (mean residence time) of Timo AIII were 18.2 ± 3.1 ng/mL, 2.3 ± 0.57 h, 4.9 ± 2.0 h, and 7.1 ± 1.4 h respectively after oral administration of Timo AIII (6.8 mg/kg) in health male SD rat (Liu Z. et al., 2013). Moreover, Timo AIII presented slow eliminated rate in the liver and caused hepatotoxicity which might result from its induction of intracellular ROS and down-regulation of bile acid transporters capabilities (Wu et al., 2014; Xie et al., 2018). And mangiferin, which is an active component in *A. asphodeloides* Bunge and presented anti-oxidant ability, attenuated Timo AIII-induced hepatotoxicity. The absorption, distribution, metabolism, and elimination (ADME) processes and toxicity of Timo AIII still need further study in both health and disease models including mice, rat, and human.

## Discussion

Timo AIII presented various pharmacological activities including anti-cancer, anti-AD, anti- diabetic mellitus, anti-colitis, anti-coagulant, and so on, while these researches were mainly conducted *in vitro*. The most effective activity of Timo AIII is anti-cancer, especially breast cancer and hepatocellular cancer, and the underlying mechanism are co-related with its anti-metastasis, anti-resistance, cytotoxicity, pro-apoptosis, and induction of cell cycle arrest, ROS, ER stress, and mitochondria dysfunction. Previous studies revealed that Timo AIII-induced autophagy might act as the role to promote tumor cell survival *in vitro*. These lack the *in vivo* evidence that explore whether inhibition of autophagy could elevate the anti-cancer effect of Timo AIII. And the autophagy induction effect of Timo AIII might contribute to other diseases, like AD and PD, in which activation of autophagy accelerates clearance of misfolding proteins. We also identified VEGFR, XIAP, BMI1, thromboxane (Tx) A2 receptor, mTOR, NF-κB, COX-2, MMPs, AChE, and so on are as the vital pharmacological targets of Timo AIII. Moreover, the anti-inflammatory effect of Timo AIII is also wealth for further studies, which accounts for its anti-AD, anti-metastasis, ant-metastasis, anti-UVB radiation, and anti-colitis activities. The toxicity, especially hepatotoxicity, and pharmacokinetics including ADME of Timo AIII are worth to investigate in diverse animal models. In conclusion, Timo AIII is potent as a compound or leading compound for further drug development while still needs in-depth studies.

## Data Availability Statement

The raw data supporting the conclusions of this article will be made available by the authors, without undue reservation, to any qualified researcher.

## Author Contributions

YL and W-RZ wrote the manuscript. W-TS, JZ, K-YZ, and QD collected the related articles and summarized the figures and tables. W-TS, X-LC, J-YT, and Z-YZ designed the study and revised the manuscript.

## Funding

This study was supported by the National Health Commission of Shanghai (GWIV-28, ZY-(2018-2020)-FWTX-8001, XHLHGG201803) and Shanghai University of Traditional Chinese Medicine (A1-N19205010302). The authors thank Mr. Hua Chaofeng from School of Management, Jiangxi Agricultural University for drawing the features of *Anemarrhena asphodeloides* Bunge according to Compendium of Materia Medica.

## Conflict of Interest

The authors declare that the research was conducted in the absence of any commercial or financial relationships that could be construed as a potential conflict of interest.

## Abbreviations

AChE, acetylcholinesterase; AMPK, 5' AMP-activated protein kinase; BCRP, breast cancer resistance protein; BMI1, B-cell-specific Moloney murine leukemia virus integration site; CDK, cyclin-dependent kinase; COX-2, Cyclooxygenase-2; IAP, inhibitor of apoptosis protein; ISVs, intersegmental vessels; MDR1, multi-drug resistance protein 1; MMP, matrix metalloproteinase; MRP1, MDR-associated protein 1; PARP, poly-(ADP ribose) polymerase; PCA, passive cutaneous anaphylaxis; PCNA, proliferating cell nuclear antigen; PRC1, polycomb repressive complex 1; SIVs, subintestinal vessels SIVs; SMC1, structural maintenance of chromosomes protein 1; TORC1 target of rapamycin complex 1; VEGFR, vascular endothelial growth factor receptor; XIAP, X-linked inhibitor of apoptosis protein.
